# Severe gastrointestinal involvement is associated with renal manifestations in pediatric IgA vasculitis: a retrospective cohort study

**DOI:** 10.1186/s12887-026-07051-7

**Published:** 2026-06-20

**Authors:** Thuy Trang Mai, Thu Ha Le

**Affiliations:** 1 Department of Nephrology and Endocrinology, Children’s Hospital 1, Ho Chi Minh City, Vietnam; 2 Department of Pediatrics, School of Medicine, University of Medicine and Pharmacy at Ho Chi Minh City, Ho Chi Minh City, Vietnam

**Keywords:** IgA vasculitis, Pediatric vasculitis, Henoch-Schönlein Purpura, Gastrointestinal involvement, Renal involvement

## Abstract

**Background:**

Gastrointestinal manifestations are common in pediatric IgA vasculitis (IgAV), but their relationship with renal involvement remains incompletely understood. Early identification of children at risk for renal complications is clinically important.

**Methods:**

We conducted a retrospective single-center cohort study including children diagnosed with IgA vasculitis between August 2023 and June 2024. Clinical characteristics, gastrointestinal manifestations, and renal outcomes were analyzed. Renal involvement was defined as hematuria and/or proteinuria by the end of follow-up, including both baseline and incident cases. Multivariable logistic regression was performed to identify factors associated with renal involvement. Sensitivity and subgroup analyses were conducted to assess the robustness of the findings.

**Results:**

Sixty-eight children with IgA vasculitis were included, of whom 44 had available follow-up data. Gastrointestinal manifestations were observed in 82.4% of patients, and among these, 55.4% had severe involvement. Among patients with follow-up data, 19/44 (43.2%) had renal involvement by the end of follow-up, including 14 cases present at baseline and 5 new-onset cases. Severe gastrointestinal involvement was associated with renal involvement in multivariable analysis (adjusted OR 4.34, 95% CI 1.48–12.65, *p* = 0.007). In sensitivity and subgroup analyses, the direction of association was consistent, although statistical significance was not maintained.

**Conclusions:**

Severe gastrointestinal involvement may be associated with an increased likelihood of early renal manifestations in pediatric IgA vasculitis. These findings suggest that closer renal monitoring may be warranted in patients presenting with severe gastrointestinal symptoms. Larger multicenter studies with longer follow-up are needed to confirm these observations.

**Supplementary Information:**

The online version contains supplementary material available at https://doi.org/10.1186/s12887-026-07051-7.

## Background

Immunoglobulin A vasculitis (IgAV), previously known as Henoch–Schönlein purpura, is a small-vessel vasculitis characterized by IgA-dominant immune deposits [1, 2]. The disease predominantly affects children and typically involves the skin, joints, gastrointestinal tract, and kidneys [2]. Among these manifestations, gastrointestinal symptoms such as abdominal pain and gastrointestinal bleeding are common and may represent early clinical features of the disease [3].

Renal involvement is one of the most clinically significant complications of IgA vasculitis because it may influence long-term outcomes [1, 3]. Although many cases follow a self-limited course, a proportion of patients develop persistent urinary abnormalities or nephritis. Early identification of children at risk for renal involvement is therefore important for timely monitoring and management.

Previous studies have suggested that severe gastrointestinal manifestations may reflect greater systemic disease activity and could be associated with renal complications [3]. However, findings remain inconsistent across populations, and data from single-center pediatric cohorts in Southeast Asia remain limited.

This study was conducted to describe gastrointestinal manifestations in children with IgA vasculitis and to evaluate their relationship with renal involvement, including the timing of renal manifestations during follow-up.

## Methods

### Study design

This was a retrospective single-center cohort study.

### Study population

The study enrolled children aged 1 month to 16 years who met the diagnostic criteria for IgA vasculitis (IgAV) and were admitted for the first time to the Nephrology-Endocrinology Department at Children’s Hospital 1 between August 2023 and June 2024 [4].

### Inclusion criteria

Patients were eligible for inclusion if they met the EULAR/PRINTO/PRES 2010 classification criteria for IgA vasculitis (Henoch–Schönlein purpura) [5]:

The diagnosis required the presence of palpable purpura or petechiae predominantly involving the lower extremities in the absence of thrombocytopenia, together with at least one of the following clinical features:

- Abdominal pain.
- Arthritis or arthralgia.
- Renal manifestations.
- Histopathological findings consistent with IgA vasculitis.

Arthritis was defined as joint swelling or limitation of motion associated with pain, whereas arthralgia referred to joint pain without objective swelling or restriction of movement.

Renal involvement was defined as the presence of proteinuria and/or hematuria, including any of the following:

- Proteinuria greater than 0.3 g per 24 h,
- Urine albumin-to-creatinine ratio > 30 mg/mmol,
- Dipstick proteinuria ≥ 2+,
- Microscopic hematuria (> 5 red blood cells per high-power field) or red blood cell casts.

The primary outcome was renal involvement by the end of follow-up, including both baseline and incident cases.

Histopathological confirmation included IgA-dominant deposition on skin biopsy or proliferative glomerulonephritis with predominant IgA deposition on kidney biopsy.

### Exclusion criteria

Patients were excluded if they had preexisting chronic systemic diseases that could affect gastrointestinal or renal manifestations, including hypertension, diabetes mellitus, inflammatory bowel disease, or coagulation disorders. Children with recurrent IgA vasculitis were also excluded from the analysis. Recurrent IgA vasculitis was defined as the reappearance of clinical symptoms (e.g., purpura, gastrointestinal or renal manifestations) after a symptom-free interval following complete resolution of the initial episode.

### Definition of gastrointestinal involvement

Gastrointestinal involvement was defined as the presence of abdominal pain or other digestive symptoms, including nausea, vomiting, diarrhea, or gastrointestinal bleeding, occurring at the time of diagnosis.

### Definition of severe gastrointestinal involvement

Severe gastrointestinal involvement was defined based on clinically significant manifestations reported in previous studies [6–8], including both symptom severity and objective complications. Specifically, severe involvement included moderate-to-severe abdominal pain assessed using the Wong–Baker FACES Pain Rating Scale, as well as the presence of gastrointestinal bleeding or the need for surgical intervention. Gastrointestinal bleeding was defined based on clinical presentation, including hematochezia or melena documented in medical records. Surgical intervention was defined as any operative procedure performed due to gastrointestinal complications. Ultrasonographic findings were based on routine abdominal ultrasound reports documented in the medical records; no standardized imaging protocol was applied because of the retrospective design.

The inclusion of abdominal pain severity reflects the clinical burden of gastrointestinal involvement in pediatric patients, as pain is often the primary symptom leading to hospitalization and clinical evaluation. This approach is consistent with prior studies evaluating clinically meaningful gastrointestinal manifestations in IgA vasculitis.

To assess the robustness of the findings, a sensitivity analysis was performed using a stricter definition of severe gastrointestinal involvement, defined as the presence of gastrointestinal bleeding or the need for surgical intervention only.

### Assessment of abdominal pain

Abdominal pain severity was assessed using the Wong–Baker FACES Pain Rating Scale (0–10), a validated tool in pediatric populations. A score > 3 was considered clinically significant and used to identify at least moderate pain severity. Pain scores were obtained retrospectively from medical records documented at the time of hospitalization.

### Classification of renal involvement

Renal involvement was categorized according to the timing of onset:

- Early renal involvement: renal manifestations detected at diagnosis or within the first month after symptom onset.
- Late renal involvement: renal manifestations occurring more than one month after disease onset within the same disease episode.

### Sample size

A total sampling approach was employed, including all eligible patients who met the inclusion criteria during the study period.

### Statistical analysis

Data were entered and analyzed using SPSS version 20.0. Categorical variables were summarized using frequency and percentage. Continuous variables were expressed as mean ± standard deviation for normally distributed data, or median with interquartile range for non-normally distributed data. Comparisons between two independent groups were made using the Independent Samples T-test for normally distributed variables and the Mann–Whitney U test for non-normally distributed variables. Associations between categorical variables were assessed using the Chi-square test, or Fisher’s exact test when expected cell counts were less than 5. To evaluate independent factors associated with renal involvement, a multivariable logistic regression analysis was performed. Variables with *p* < 0.1 in univariate analysis or clinically relevant factors (age, sex, and severe gastrointestinal involvement) were included in the model. Odds ratios (ORs) with 95% confidence intervals (CIs) were calculated.

## Results

A total of 68 pediatric patients with IgA vasculitis were included in the baseline analysis. Among them, 24 patients were lost to follow-up after hospital discharge and were excluded from longitudinal outcome assessment. Consequently, renal outcomes were evaluated in 44 patients with available follow-up data. The median follow-up duration was 3 months (range 1 to 6 months) (Table 1).

**Table 1:** Baseline characteristics of the study population (*n* = 68)

Variable	GI group (*n* = 56)	Non-GI group (*n* = 12)	*p*-value
Age (years, mean ± SD)	7.51 ± 3.14	9.08 ± 2.64	0.11
Sex (male, n (%))	36 (64.3%)	9 (75.0%)	0.47
Baseline renal involvement (n (%))	16 (28.6%)	4 (33.3%)	0.74

Among patients with gastrointestinal involvement, 55.4% had severe manifestations. There were no significant differences in baseline characteristics between patients with and without gastrointestinal involvement. A comparison of baseline characteristics between patients with and without follow-up data showed no statistically significant differences in age, sex, gastrointestinal involvement, severity of gastrointestinal manifestations, or baseline renal involvement (Table S1). These findings suggest that loss to follow-up is unlikely to have introduced substantial selection bias (Table 2).

**Table 2:** Clinical characteristics of gastrointestinal manifestations (*n* = 56)

Variable	Total (*n* = 56)
Abdominal pain (n, %)	56 (100%)
Pain score > 3 (n, %)	27 (48.2%)
GI bleeding (n, %)	14 (25.0%)
Surgery (n, %)	1 (1.7%)
Ultrasound findings (n, %)	25 (44.6%)

Abdominal pain severity was assessed using the Wong–Baker FACES Pain Rating Scale (0–10), with scores > 3 considered clinically significant.

Gastrointestinal bleeding was the most clinically significant complication, although less frequent than abdominal pain. Only one patient required surgical intervention due to gastrointestinal complications.

Gastrointestinal manifestations were primarily managed with glucocorticoids. Intravenous glucocorticoids were administered in the majority of patients (52/56, 92.9%), with a median dose of approximately 2 mg/kg in both groups. The median duration of intravenous glucocorticoid therapy was 4 days (IQR 2.5–6.0) in patients with severe gastrointestinal involvement and 3 days (IQR 3–4) in those without. The median time to resolution of abdominal pain after glucocorticoid initiation was significantly longer in the severe group compared to the non-severe group (2 [1–2.5] vs. 1 [1–2] days, *p* = 0.034). Similarly, the duration of hospitalization was longer in patients with severe gastrointestinal involvement (6 [4–8] vs. 5 [3.3–6] days, *p* = 0.020) Table 3.

**Table 3:** Renal manifestations in the study population

Variable/Value	*n* = 68 (%)
Baseline renal involvement	20 (29.4%)
New-onset renal involvement	5/30* (16.7%)
Hematuria	15 (22.0%)
Proteinuria	15 (22.0%)
Nephrotic-range proteinuria	0 (0%)
Biopsy performed	4 (5.9%)

*Among patients without renal involvement at baseline and with available follow-up data

**Fig. 1:**
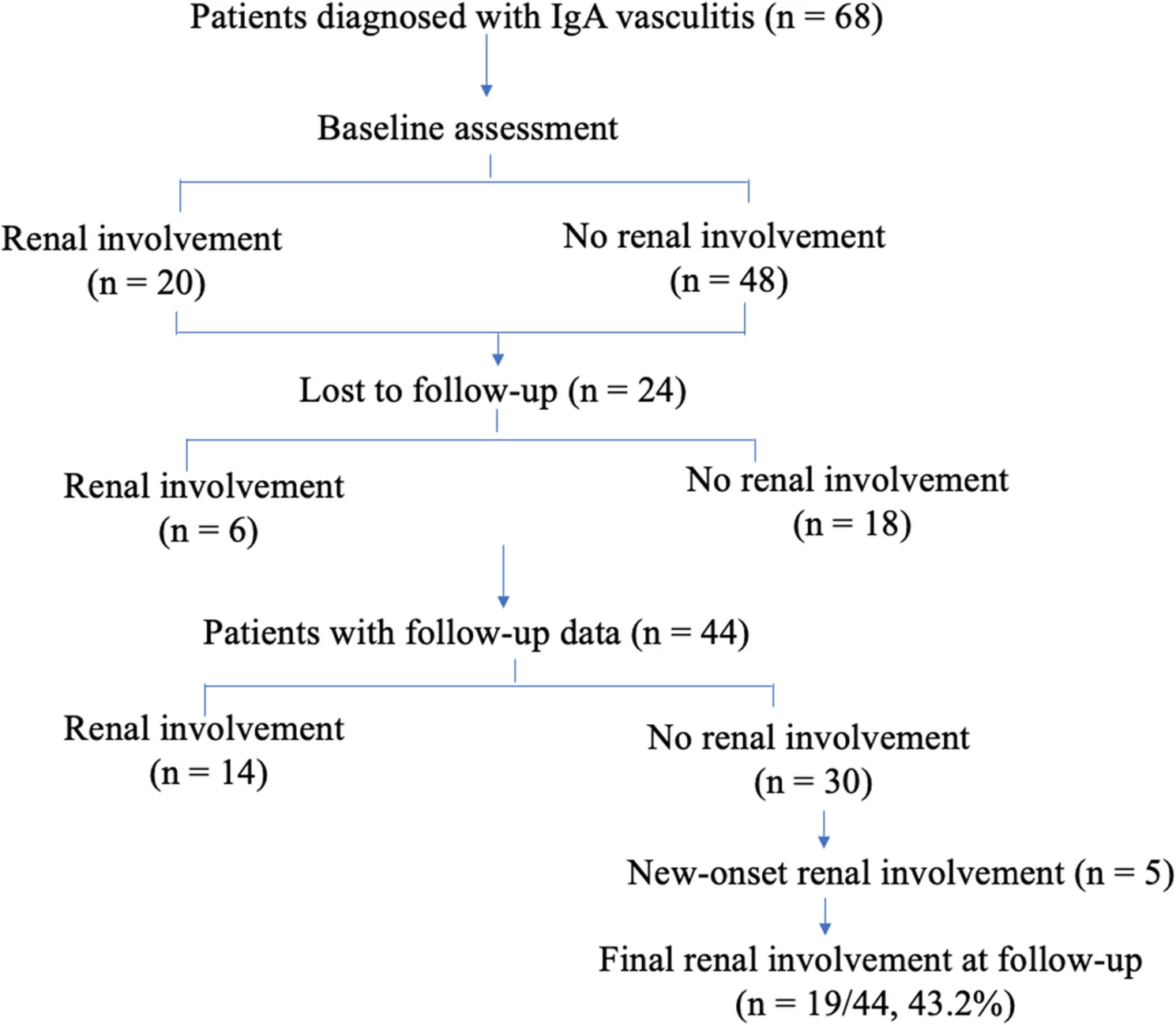
Flow diagram of patient inclusion and renal outcome assessment. A total of 68 patients were included at baseline. Twenty-four patients were lost to follow-up, leaving 44 patients for longitudinal analysis. Among these, 14 had renal involvement at baseline and 5 additional patients developed new-onset renal involvement during follow-up, resulting in an overall renal involvement rate of 43.2% among patients with follow-up data.

Renal manifestations were generally mild in our cohort. Most patients presented with microscopic hematuria and/or low-grade proteinuria. No cases of nephrotic-range proteinuria, nephrotic syndrome, or renal failure were observed. Kidney biopsy was performed in 4 patients based on clinical indications, mainly persistent urinary abnormalities. All biopsy specimens showed IgA-dominant deposition consistent with IgA vasculitis nephritis. Detailed histopathological grading (e.g., ISKDC classification) was not consistently available, and no crescentic lesions were reported. Due to the small number of cases, no formal comparison between gastrointestinal severity and histopathological findings was performed (Fig. 1).

 Severe gastrointestinal involvement was independently associated with an increased risk of renal involvement in multivariable analysis (adjusted OR 4.34, 95% CI: 1.48–12.65, *p* = 0.007) (Tables 4).

**Table 4:** Association between severe gastrointestinal involvement and renal outcome

Variable	Crude OR (95% CI)	Adjusted OR (95% CI)	*p*-value
Severe GI	4.40 (1.53**–**12.64)	4.34 (1.48–12.65)	0.007
Age	0.97 (0.83**–**1.14)	0.96 (0.81**–**1.15)	0.72
Sex	1.52 (0.52**–**4.42)	1.19 (0.37**–**3.79)	0.76

In sensitivity analysis using a stricter definition of severe gastrointestinal involvement, defined as gastrointestinal bleeding or the need for surgical intervention, the association with renal involvement was attenuated and did not reach statistical significance (OR = 2.37, 95% CI: 0.71–7.85, *p* = 0.15).

In subgroup analysis restricted to patients without renal involvement at baseline, severe gastrointestinal involvement was associated with an increased risk of incident renal involvement (OR = 3.63, 95% CI: 0.44–29.91), although the association did not reach statistical significance (*p* = 0.23).

## Discussion

This study demonstrated that severe gastrointestinal involvement was associated with an increased likelihood of renal manifestations in pediatric IgA vasculitis, even after adjustment for potential confounders. In sensitivity and subgroup analyses, although the magnitude of the association remained elevated, statistical significance was not consistently achieved, likely due to the limited number of events. Therefore, these findings should be interpreted with caution.

The observed association is biologically plausible. IgA vasculitis is a systemic small-vessel vasculitis, and more severe gastrointestinal involvement may reflect a higher degree of systemic vascular inflammation, which in turn may predispose to renal injury through shared pathogenic mechanisms such as IgA deposition and endothelial damage. Several previous studies have also suggested an association between severe gastrointestinal manifestations and renal involvement, although findings remain inconsistent across populations [3, 6–9]. These discrepancies may be explained by differences in study design, sample size, and definitions of gastrointestinal severity.

In our cohort, gastrointestinal involvement was observed in a large proportion of patients, with a relatively high rate of severe manifestations compared with previous reports [6, 7, 10]. This may be partly explained by referral bias, as patients admitted to a tertiary center are more likely to present with more severe clinical features. Despite this, the pattern of gastrointestinal manifestations in our study was consistent with prior literature, with abdominal pain being the most common symptom and gastrointestinal bleeding representing the most clinically significant complication [7, 10, 11].

In our cohort, gastrointestinal manifestations were primarily managed with glucocorticoids, which were administered in the majority of patients. Children with severe gastrointestinal involvement had a longer time to resolution of abdominal pain and a longer duration of hospitalization compared with those without severe manifestations. These findings are consistent with the clinical observation that more severe gastrointestinal involvement reflects a higher disease burden and may require more intensive management.

However, the potential impact of glucocorticoid therapy on renal outcomes should be considered when interpreting the association observed in this study. Due to the retrospective design, the initiation, dose, and duration of glucocorticoid treatment were not standardized and were based on clinical judgment. As a result, treatment-related confounding cannot be excluded. It is possible that patients with more severe gastrointestinal manifestations were more likely to receive glucocorticoids, which may have influenced the occurrence or detection of renal involvement.

Previous studies have reported mixed findings regarding the role of glucocorticoids in preventing renal complications in IgA vasculitis. While glucocorticoids are commonly used to manage severe gastrointestinal symptoms, their effect on long-term renal outcomes remains uncertain [6, 9]. Therefore, the association between severe gastrointestinal involvement and renal manifestations observed in this study should be interpreted in the context of potential treatment-related confounding.

Renal involvement in this study was generally mild, with most patients presenting with microscopic hematuria and/or low-grade proteinuria. No cases of nephrotic syndrome or renal failure were observed during follow-up. The overall rate of renal involvement at the end of follow-up (43.2%) was within the upper range reported in previous studies. Consistent with prior literature, most renal manifestations occurred early in the disease course, typically within the first month after onset [3, 12–14]. Therefore, the findings of this study primarily reflect early renal involvement rather than long-term renal outcomes.

In sensitivity analysis using a stricter definition of severe gastrointestinal involvement (limited to gastrointestinal bleeding or surgical intervention), the association remained directionally consistent but did not reach statistical significance. Similarly, in subgroup analysis restricted to patients without renal involvement at baseline, the association remained elevated but was not statistically significant. These findings suggest that the study may be underpowered to detect statistically significant differences across all analyses, although the consistency in effect direction supports a potential relationship.

Several limitations should be considered when interpreting these findings. First, this was a single-center retrospective study with a relatively small sample size, which may limit the generalizability of the results. Second, a substantial proportion of patients were lost to follow-up, which may introduce selection bias. Although no statistically significant differences were observed between patients with and without follow-up data, residual bias cannot be excluded. Third, the limited number of renal events may affect the stability of the multivariable model and increase the risk of overfitting; therefore, the results should be considered exploratory.

In addition, renal involvement was defined based on the presence of hematuria and/or proteinuria, which may capture mild or transient abnormalities and may not fully reflect clinically significant renal disease. The relatively short follow-up duration further limits the ability to assess persistent or late-onset renal involvement. As such, the findings of this study primarily reflect early renal manifestations rather than long-term renal outcomes.

Despite these limitations, this study provides real-world data on pediatric IgA vasculitis in a Southeast Asian setting, where such data remain limited. The use of multivariable analysis, along with sensitivity and subgroup analyses, strengthens the validity of the findings within the constraints of the study design.

From a clinical perspective, children presenting with severe gastrointestinal manifestations may represent a subgroup at higher risk of early renal involvement. These findings highlight the importance of careful and repeated renal monitoring in the early phase of the disease, particularly within the first weeks after diagnosis.

Further multicenter studies with larger sample sizes and longer follow-up durations are needed to confirm these findings and to better characterize the relationship between gastrointestinal severity and long-term renal outcomes in pediatric IgA vasculitis.

## Conclusion

Severe gastrointestinal involvement may be associated with an increased likelihood of renal involvement in pediatric IgA vasculitis, particularly during the early phase of the disease. These findings support the consideration of closer renal monitoring in children presenting with severe gastrointestinal manifestations. However, given the retrospective design, limited sample size, and short follow-up duration, the results should be interpreted with caution. Further multicenter studies with larger sample sizes and longer follow-up are required to better define the relationship between gastrointestinal severity and renal outcomes.

## Supplementary Information

Supplementary Material 1

## Data Availability

The datasets used and/or analyzed during the current study are available from the corresponding author on reasonable request.

## Abbreviations

IgAV: IgA vasculitis
GI: Gastrointestinal
