# Supplementary material for: Severe gastrointestinal involvement is associated with renal manifestations in pediatric IgA vasculitis: a retrospective cohort study
Source: BMC Pediatr. 2026 Jun 20;26:1099. doi: 10.1186/s12887-026-07051-7 (PMC13729626; doi:10.1186/s12887-026-07051-7)
Supplement: Supplementary file 1 [file 12887_2026_7051_MOESM1_ESM.docx]

# Table S1. Baseline characteristics between patients with and without follow-up

| Variable | Follow-up (n = 44) | Lost (n = 24) | p-value |
| --- | --- | --- | --- |
| Age (years, mean ± SD) | 7.8 ± 3.2 | 7.7 ± 2.9 | 0.96 |
| Male sex, n (%) | 27 (61.4%) | 18 (75.0%) | 0.25 |
| GI involvement, n (%) | 35 (79.5%) | 21 (87.5%) | 0.51 |
| Severe GI, n (%) | 22 (50.0%) | 9 (37.5%) | 0.32 |
| Baseline renal involvement, n (%) | 14 (31.8%) | 6 (25.0%) | 0.55 |
